# New Internet-Based Warfarin Anticoagulation Management Approach After Mechanical Heart Valve Replacement: Prospective, Multicenter, Randomized Controlled Trial

**DOI:** 10.2196/29529

**Published:** 2021-08-13

**Authors:** Zhihui Zhu, Chenyu Li, Jinglun Shen, Kaisheng Wu, Yuehuan Li, Kun Liu, Fan Zhang, Zhenhua Zhang, Yan Li, Jie Han, Ying Qin, Yu Yang, Guangpu Fan, Huajun Zhang, Zheng Ding, Dong Xu, Yu Chen, Yingli Zheng, Zhe Zheng, Xu Meng, Haibo Zhang

**Affiliations:** 1 Department of Cardiac Surgery Beijing Anzhen Hospital Capital Medical University Beijing China; 2 Ludwig Maximilian University of Munich Munich Germany; 3 Renal Division Department of Medicine IV Ludwig Maximilian University of Munich Munich Germany; 4 Department of Cardiovascular Surgery Beijing Luhe Hospital, Capital Medical University Beijing China; 5 Department of Cardiovascular Surgery Beijing Xuanwu Hospital Beijing China; 6 Department of Cardiovascular Surgery Peking University People’s Hospital Beijing China; 7 Department of Cardiovascular Surgery PLA General Hospital Beijing China; 8 Department of Pharmacy Fuwai Hospital, National Center for Cardiovascular Diseases Chinese Academy of Medical Sciences and Peking Union Medical College Beijing China; 9 Department of Cardiovascular Surgery Fuwai Hospital, National Center for Cardiovascular Diseases Chinese Academy of Medical Sciences and Peking Union Medical College Beijing China

**Keywords:** RCT, warfarin, telemedicine, TTR, complication

## Abstract

**Background:**

Mechanical heart valve replacement (MHVR) is an effective method for the treatment of severe heart valve disease; however, it subjects patient to lifelong warfarin therapy after MHVR with the attendant risk of bleeding and thrombosis. Whether internet-based warfarin management reduces complications and improves patient quality of life remains unknown.

**Objective:**

This study aimed to compare the effects of internet-based warfarin management and the conventional approach in patients who received MHVR in order to provide evidence regarding alternative strategies for long-term anticoagulation.

**Methods:**

This was a prospective, multicenter, randomized, open-label, controlled clinical trial with a 1-year follow-up. Patients who needed long-term warfarin anticoagulation after MHVR were enrolled and then randomly divided into conventional and internet-based management groups. The percentage of time in the therapeutic range (TTR) was used as the primary outcome, while bleeding, thrombosis, and other events were the secondary outcomes.

**Results:**

A total of 721 patients were enrolled. The baseline characteristics did not reach statistical differences between the 2 groups, suggesting the random assignment was successful. As a result, the internet-based group showed a significantly higher TTR (mean 0.53, SD 0.24 vs mean 0.46, SD 0.21; *P*<.001) and fraction of time in the therapeutic range (mean 0.48, SD 0.22 vs mean 0.42, SD 0.19; *P*<.001) than did those in the conventional group. Furthermore, as expected, the anticoagulation complications, including the bleeding and embolic events had a lower frequency in the internet-based group than in the conventional group (6.94% vs 12.74%; *P*=.01). Logistic regression showed that internet-based management increased the TTR by 7% (odds ratio [OR] 1.07, 95% CI 1.05-1.09; *P*<.001) and reduced the bleeding and embolic risk by 6% (OR 0.94, 95% CI 0.92-0.96; *P*=.01). Moreover, low TTR was found to be a risk factor for bleeding and embolic events (OR 0.87, 95% CI 0.83-0.91; *P*=.005).

**Conclusions:**

The internet-based warfarin management is superior to the conventional method, as it can reduce the anticoagulation complications in patients who receive long-term warfarin anticoagulation after MHVR.

**Trial Registration:**

Chinese Clinical Trial Registry ChiCTR1800016204; http://www.chictr.org.cn/showproj.aspx?proj=27518

**International Registered Report Identifier (IRRID):**

RR2-10.1136/bmjopen-2019-032949

## Introduction

Heart valve replacement is recommended for patients with severe heart valve disease and is performed in several hundred thousand patients worldwide each year [1]. As a replacement valve for humans, a mechanical valve is more durable than a bioprosthetic valve but requires lifelong anticoagulation therapy [2]. Over 98% of young Chinese patients who need heart valve replacement receive mechanical valves [3] but face the risk of thrombosis on the valves and ensuing embolism [4]; thus, lifelong anticoagulation is required and recommended by guidelines [5]. Warfarin, as an anticoagulation drug, is widely used in the prevention of various thromboembolic events [6], but it is not easy to control due to the narrow therapeutic range and patients’ heterogeneity. Therefore, the dosage of warfarin needs to be adjusted accordingly by the international normalized ratio (INR) [7,8]. Time in therapeutic range (TTR), as the percentage of time the patient’s INR is within the target range, has been used in clinical research to measure the adequacy of warfarin therapy and shows a significant correlation with anticoagulation outcomes [9,10]. Well-managed warfarin anticoagulation is effective in reducing complications, such as bleeding and thrombosis, in patients with a mechanical heart valve [11]. However, in clinical practice, patients’ anticoagulation management is not conducted in ideal fashion.

The conventional way to manage anticoagulation is face to face [12], but the quality of this approach depends highly on the patients’ compliance, resulting in difficulty in management and increased risk of adverse events [13,14]. Chinese cardiac surgeons tend to focus more on inpatient treatment, but follow-up is often ignored, especially in central and western China, where there is a lack of professional cardiac surgery–related health care [15]. Furthermore, the extensive follow-up required for warfarin management adds to the workload of specialist hospitals [16,17]. Therefore, current warfarin management needs to be urgently improved.

The internet provides fast and convenient communication, with integration into health care delivery systems presenting exciting opportunities for improving care promotion, disease prevention, and value-based clinical medicine [18]. Previous studies have reported several online warfarin management systems and have demonstrated their benefits for patients after heart valve surgery [19-21]. However, the capacity of these telemedicine apps to improve the quality of anticoagulation remains unclear, and carefully designed prospective trials are needed to provide reliable evidence. Therefore, in this large scale, prospective, multicenter, randomized controlled trial, we aimed to study if internet-based warfarin management increases TTR and reduces the risk of anticoagulation complications in patients after mechanical heart valve replacement. Our study is the first randomized controlled trial to explore the effectiveness of internet-based warfarin anticoagulation management in patients after mechanical heart valve replacement.

## Methods

### Study Design

**Figure 1:**
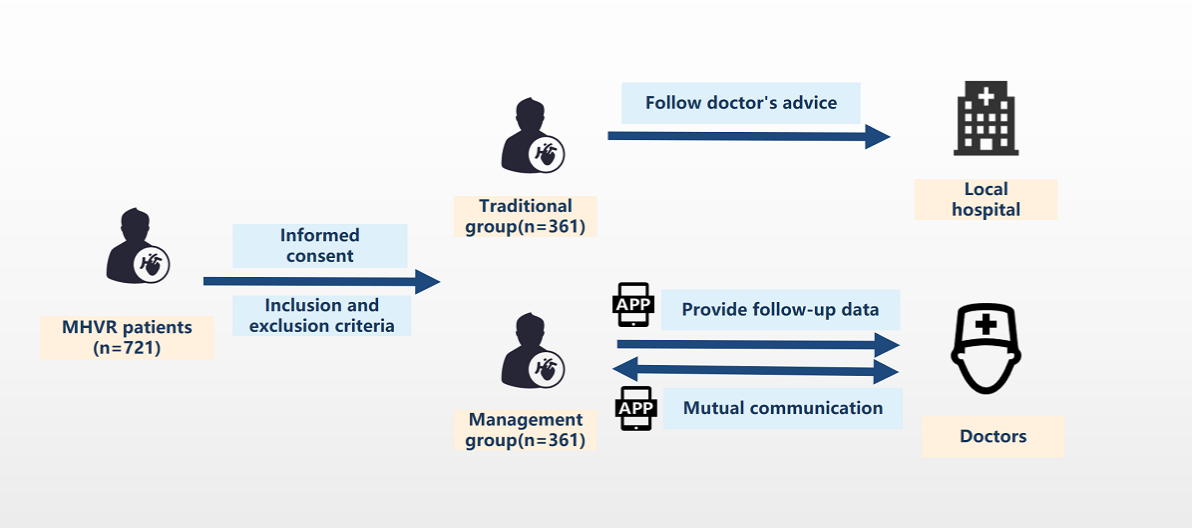
Patient flow chart. MHVR mechanical heart valve replacement.

The clinical trial design has been described previously [16]. In brief, the study was a prospective, multicenter, randomized, open-label, controlled trial. The patient flow chart is shown in Figure 1. The project was organized and implemented by Beijing Anshan Hospital affiliated with Capital Medical University. Five top cardiac centers in China participated in the clinical trial. The Peking University Clinical Research Institute has created a data committee to evaluate the data quality and supervise data collecting.

All participants provided written informed consent. The study protocol was approved by the Ethics Committee of Beijing Anshan Hospital, Capital Medical University, and all other centers accepted the central ethics approval or obtained local approval by internal ethics committees.

### Participants

Inclusion criteria were as follows: patients had a normal psychological state and were aged 18 to 65 years, patients or guardians agreed to the study plan and signed the informed consent form, long-term anticoagulation was required after mechanical heart valve replacement, and patients and their families could effectively operate smartphones. The exclusion criteria were the following: patients with emergency surgery; patients treated with cardiac bypass surgery; patients with severe chronic diseases, such as malignant tumors, hepatic cirrhosis, diabetes, cerebral hemorrhage (cerebral infarction), convalescence, thyroid dysfunction, and respiratory failure; patients with severe renal insufficiency (endogenous creatinine clearance rate ≤20 mL/min); patients with severe hepatic insufficiency (Child-Pugh ≥10); patients with severe heart failure (cardiac function New York Heart Association grade IV); patients with postoperative infective endocarditis; patients with severe postoperative complications and poor prognosis; and patients who were unable to comply with this study.

### Intervention

In our study, the conventional management was conducted by laboratory testing and drug dose adjustment at the hospital as instructed by a doctor, while internet-based management was performed via a mobile user interface medical network follow-up platform. All patients in both groups received training and education on warfarin anticoagulation before discharge, which included information on drug interactions, diet, compliance with dosing, and the importance of INR testing.

In the conventional anticoagulation management model, patients underwent INR tests at the local hospital. After reviewing the laboratory results, the outpatient cardiologist evaluated the INR results and decided whether to adjust the warfarin dosage until the next patient visit. Patients were asked to record the results of every examination and note the physical symptoms related to warfarin anticoagulation, such as bleeding and embolism, to prepare for follow-up discharge.

In the early stages of the study, we developed an internet-based follow-up system for clinical use after cardiac surgery [22]. In the new warfarin management group, online registration information was generated by researchers and sent to patients, who completed the rest of the follow-up registration. The app software for the internet-based follow-up management system has been described previously [16]. All patients and their families attended at least 3 follow-up training sessions, including watching an educational video and installation and simulation of the follow-up software and mobile apps. Patients were asked to upload the INR and other laboratory test results to the apps.

### Randomization, Blinding, and Grouping

The central stratified randomization method was used in this study. Patients who met the inclusion and not exclusion criteria and consented to participate in this study were randomized in a 1:1 ratio into the conventional and internet-based management groups. Patients were aware of their grouping, whereas the researchers conducting the end point evaluations were blinded to the groupings.

### Anticoagulation Treatment Strategies and Follow-Up

The INR was measured every 3 to 5 days for 1 month after discharge. After the patient’s INR results stabilized, the INR was measured every 10 to 14 days from 1 to 3 months after discharge, once a month from 3 to 6 months, and once every 2 months from 6 to 12 months after discharge. The target INR window ranged from 1.8 to 2.5 (1.8-2.2 for aortic valve replacement, 2.0-2.5 for mitral valve replacement, and 2.0-2.5 for double valve replacement).

Patients were followed up at 3 months (30 days before and after), 6 months (30 days before and after), 9 months (30 days before and after), and 12 months (30 days before and after) after discharge.

At each follow-up point, patients in the traditional group were asked to bring their original hospitalization records and all medical examination results after discharge, and the researchers completed the case report form. Patients were followed up by telephone if they could not return to the hospital at the scheduled time points.

For patients in the new warfarin group, the researchers checked the data results uploaded by the patients in the new follow-up system app and completed the case report form at the follow-up time points.

### End Point

The primary end point of this study was the TTR. The TTR was measured by Roosendaal linear interpolation [10]. The secondary end point was the incidence of anticoagulation-related embolism and bleeding. Embolism includes any embolic event that occurs in the absence of infection after the immediate perioperative period. Embolism may manifest as a neurologic event or a noncerebral embolic event. Severe bleeding associated with anticoagulation includes any episode of major internal or external bleeding that causes death, hospitalization, or permanent injury or that necessitates transfusion. General bleeding related to anticoagulation refers to bleeding of the nasal cavity and gums, skin ecchymosis, menorrhea, hematuria, melena, and so on.

### Statistical Analyses

Continuous variables are expressed as the mean and SD or median and IQR; meanwhile, categorical variables are expressed as frequencies and percentages. Baseline characteristics were compared between the 2 groups by the chi-square, *t*, and Mann-Whitney tests as appropriate. The Mann-Whitney test was used to determine the difference in the TTR between the conventional anticoagulation management model group and the internet-based anticoagulation model group. The incidence of adverse events (bleeding or thrombosis) was compared by the chi-square test. A 2-sided *P* value <0.05 was regarded as statistically significant. All analyses were performed using R version 3.4.2 (The R Foundation).

## Results

### Characteristics of the Patients

A total of 721 patients from 5 top Chinese cardiovascular centers were enrolled between July 2018 and September 2019 and then randomly assigned to a conventional group (n=361) or an internet-based warfarin management group (n=360). The average ages of the conventional and internet-based group were 49.59 (SD 9.46) and 50.6 (SD 9.65), respectively (*P*=0.16); the percentage of female patients in the 2 groups was 38.9% (140/360) and 39.1% (141/361), respectively (*P*=0.96). The other baseline characteristics also did not reach statistical differences between the 2 groups (Table 1), suggesting the patients’ random assignment was successful.

**Table 1:** Patient characteristics.

Characteristics				Internet-based group (n=360)		Conventional group (n=361)		*P* value	
**Demographics**									
	Age (years), mean (SD^a^)			49.59 (9.46)		50.6 (9.65)		.16	
	Female sex, n (%)			140 (38.89)		141 (39.06)		.96	
**Risk factors**									
	Hypertension, n (%)			92 (25.56)		99 (27.42)		.57	
	Atrial fibrillation, n (%)			69 (19.17)		71 (19.67)		.87	
	Angina, n (%)			11 (3.06)		13 (3.6)		.68	
	**Smoking status**								.55
		Smokers, n (%)	67 (18.61)		63 (17.45)				
		Nonsmokers, n (%)	224 (62.22)		233 (64.54)				
		Former smokers, n (%)	69 (19.17)		65 (18.01)				
**Clinical data, mean (SD)**									
	BMI^b^, kg/m^2^			24.23 (3.34)		24.52 (3.52)		.25	
	Preoperative resting heart rate (times/minute)			78.46 (15.41)		78.73 (15.17)		.81	
	Preoperative EF^c^ (%)			61.18 (7.03)		60.88 (7.59)		.58	
	Preoperative platelet (10^9/L)			201.57 (55.97)		201.35 (56.45)		.96	
	Preoperative ALT^d^ (U/L)			25.21 (23.71)		25.47 (20.56)		.88	
	Preoperative AST^e^ (U/L)			27.3 (22.28)		27.49 (16.34)		.90	
	Preoperative BUN^f^ (mmol/L)			6.51 (2.29)		6.54 (2.23)		.86	
	Preoperative Cr^g^ (umol/L)			79.73(19.76)		79.39 (20.77)		.82	
	Postoperative resting heart rate (times/minute)			87.51(15.81)		87.02 (15.89)		.67	
	Postoperative ALT (U/L)			44.77 (46.03)		40.9 (36.5)		.21	
	Postoperative AST (U/L)			38.57 (26)		35.84 (22.96)		.14	
	Postoperative BUN (mmol/L)			7.85 (3.27)		7.88 (3.33)		.89	
	Postoperative Cr (umol/L)			74.37 (25.72)		76.19 (23.61)		.32	

^a^SD: standard deviation.

^b^BMI: body mass index.

^c^EF: ejection fraction.

^d^ALT: alanine transaminase.

^e^AST: aspartate transaminase.

^f^BUN: blood urea nitrogen.

^g^Cr: creatinine.

### Internet-Based Warfarin Management Increased TTR

In order to study the effect of internet-based management on warfarin anticoagulation, we used TTR as the primary end point. The TTR was significantly higher in the internet-based group than in the conventional group (internet: mean 0.53, SD 0.24; conventional: mean 0.46, SD 0.21; *P*<.001). Of the 360 patients in the internet-based group, the number of patients with a TTR in the range of 0%-30%, 30%-60%, and 60%-100% was 61 (16.94%), 156 (43.33%), and 143 (39.72%), respectively; meanwhile, of the 361 patients in the conventional group, the number was 77 (21.33%), 189 (52.35%), and 95 (26.32%), respectively. As expected, the internet-based group yielded a higher fraction of TTR than did the traditional group (internet: mean 0.48, SD 0.22; conventional: mean 0.42, SD 0.19; *P*<.001). Logistic regression indicated that the internet-based management increased the TTR by 7% (odds ratio [OR] 1.07, 95% CI 1.05-1.09; *P*<.001; Table 2). Our results suggest that internet-based warfarin management is better than the conventional method according to increased TTR, while the mean INR values (internet: mean 2.13, SD 0.87; conventional: mean 2.14, SD 1.10; *P*=0.87) were not different between the 2 groups (Table 3).

**Table 2:** Logistic regression of time in the therapeutic range.

Factor	β	OR^b^ (95% CI)	*t*	*P* value
Age	0 (0 to 0)	1 (1 to 1)	1.35	.18
Female sex	–0.01 (–0.03 to 0.01)	0.99 (0.98 to 1.01)	–0.44	.66
Hypertension	0.05 (0.03 to 0.07)	1.05 (1.03 to 1.07)	2.74	.01
Atrial fibrillation	–0.06 (–0.08 to –0.03)	0.95 (0.93 to 0.97)	–2.56	.01
Angina	0.06 (0.01 to 0.11)	1.06 (1.01 to 1.11)	1.25	.21
Smoking status	0.01 (0 to 0.02)	1.01 (1 to 1.02)	1.06	.29
BMI^c^	0.01 (0 to 0.01)	1.01 (1 to 1.01)	2.71	.01
Preoperative resting heart rate	0 (0 to 0)	1 (1 to 1)	–0.22	.83
Preoperative EF^d^	0 (0 to 0)	1 (1 to 1)	–0.38	.70
Preoperative platelet	0 (0 to 0)	1 (1 to 1)	–0.37	.71
Preoperative ALT^e^	0 (0 to 0)	1 (1 to 1)	–0.73	.46
Preoperative AST^f^	0 (0 to 0)	1 (1 to 1)	–0.85	.39
Preoperative BUN^g^	0 (0 to 0)	1 (1 to 1)	–0.21	.83
Preoperative Cr^h^	0 (0 to 0)	1 (1 to 1)	–2.29	.02
Postoperative resting heart rate	0 (0 to 0)	1 (1 to 1)	–0.29	.77
Postoperative ALT	0 (0 to 0)	1 (1 to 1)	1.48	.14
Postoperative AST	0 (0 to 0)	1 (1 to 1)	1.46	.14
Postoperative BUN	0.01 (0.01 to 0.01)	1.01 (1.01 to 1.01)	3.02	.002
Postoperative Cr	0 (0 to 0)	1 (1 to 1)	–1.29	.20
Group	0.07 (0.05 to 0.08)	1.07 (1.05 to 1.09)	3.96	<.001
FTTR^i^	0.97 (0.95 to 0.99)	2.63 (2.57 to 2.69)	4.46	<.001
General bleeding event	–0.08 (–0.11 to –0.05)	0.92 (0.89 to 0.95)	–2.78	.01
Severe bleeding event	–0.11 (–0.21 to –0.02)	0.89 (0.81 to 0.98)	–1.19	.23
All bleeding events	–0.09 (–0.12 to –0.06)	0.92 (0.89 to 0.94)	–3.05	.002
Neurologic embolic event	0.08(–0.08 to 0.24)	1.08 (0.92 to 1.27)	0.49	.62
Noncerebral embolic event	0.09 (–0.14 to 0.32)	1.09 (0.87 to 1.38)	0.39	.70
All embolic events	0.08 (–0.05 to 0.22)	1.09 (0.95 to 1.24)	0.62	.53
All bleeding and embolic events	–0.08 (–0.11 to –0.05)	0.92 (0.9 to 0.95)	–2.85	.005
Revisit to hospital due to secondary end point	–0.05 (–0.12 to 0.02)	0.95 (0.88 to 1.02)	–0.69	.49
Death due to secondary end point	0.03 (–0.14 to 0.19)	1.03 (0.87 to 1.21)	0.16	.87

^a^TTR: time in the therapeutic range.

^b^OR: odds ratio.

^c^BMI: body mass index.

^d^EF: ejection fraction.

^e^ALT: alanine transaminase.

^f^AST: aspartate transaminase.

^g^BUN: blood urea nitrogen.

^h^Cr: creatinine.

^i^FTTR: fraction of time in therapeutic range.

**Table 3:** Primary end point.

End point	Internet-based group (n=360)	Traditional group (n=361)	*P* value
INR^a^, mean (SD^b^)	2.13 (0.87)	2.14 (1.10)	.87
TTR^c^, mean (SD)	0.53 (0.24)	0.46 (0.21)	<.001
0%-30%, n (%)	61 (16.94)	77 (21.33)	N/A^d^
30%-60%, n (%)	156 (43.33)	189 (52.35)	N/A
60%-100%, n (%)	143 (39.72)	95 (26.32)	N/A
FTTR^e^, mean (SD)	0.48 (0.22)	0.42 (0.19)	<.001

^a^INR: international normalized ratio.

^b^SD: standard deviation.

^c^TTR: time in the therapeutic range.

^d^N/A: not applicable.

^e^FTTR: fraction of time in therapeutic range.

### Internet-Based Warfarin Management Reduced the Risk of Adverse Events

A lower risk of stroke and bleeding can reached by maximizing the TTR. We further used the bleeding and embolic events as a secondary end point to investigate the difference between the internet-based and conventional groups. The incidence of all bleeding and embolic events (6.94% vs 12.74%; *P=*.009) was lower in the internet-based group than in the conventional group (Table 4). Logistic regression showed that internet-based management reduced the bleeding and embolic risk by 6% (OR 0.94, 95% CI 0.92-0.96; *P=*.01). Moreover, low TTR was a risk factor for bleeding and embolic events (OR 0.87, 95% CI 0.83-0.91; *P=*.005; Table 5). The results showed that internet-based warfarin management not only increased TTR but also reduced the risk of adverse events of warfarin anticoagulation.

**Table 4:** Adverse events.

Adverse event	Internet-based group (n=360)	Traditional group (n=361)	*P* value
General bleeding event, n (%)	22(6.11)	40(11.08)	.02
Severe bleeding event, n (%)	2(0.56)	4(1.11)	.41
All bleeding events, n (%)	24(6.67)	44(12.19)	.01
Neurologic embolic event, n (%)	1(0.28)	1(0.28)	>.99
Noncerebral embolic event, n (%)	0(0)	1(0.28)	.32
All embolic events, n (%)	1(0.28)	2(0.55)	.56
All bleeding and embolic events, n (%)	25(6.94)	46(12.74)	.01
Revisit to hospital due to secondary end point, n (%)	4(1.11)	6(1.66)	.53
Death due to secondary end point, n (%)	0(0)	2(0.55)	.87

**Table 5:** Logistic regression of all bleeding and embolic events.

Characteristic	β	OR^a^ (95% CI)	*t*	*P* value
Age	0 (0 to 0)	1 (1 to 1)	0.51	.61
Female sex	–0.02 (–0.04 to 0)	0.98 (0.96 to 1)	–0.94	.35
Hypertension	0.02 (0 to 0.05)	1.02 (1 to 1.05)	0.90	.37
Atrial fibrillation	0.04 (0.01 to 0.07)	1.04 (1.01 to 1.07)	1.33	.18
Angina	0.11 (0.05 to 0.18)	1.12 (1.05 to 1.19)	1.84	.07
Smoking status	0.03 (0.01 to 0.04)	1.03 (1.01 to 1.04)	2.04	.04
BMI^b^	0 (0 to 0)	1 (1 to 1)	–0.53	.60
Preoperative resting heart rate	0 (0 to 0)	1 (1 to 1)	–0.31	.75
Preoperative EF^c^	0 (0 to 0)	1 (1 to 1)	–0.99	.32
Preoperative platelet	0 (0 to 0)	1 (1 to 1)	–1.18	.24
Preoperative ALT^d^	0 (0 to 0)	1 (1 to 1)	0.03	.98
Preoperative AST^e^	0 (0 to 0)	1 (1 to 1)	0.67	.51
Preoperative BUN^f^	0.01 (0 to 0.01)	1.01 (1 to 1.01)	1.81	.07
Preoperative Cr^g^	0 (0 to 0)	1 (1 to 1)	1.70	.09
Postoperative resting heart rate	0 (0 to 0)	1 (1 to 1)	–1.06	.29
Postoperative ALT	0 (0 to 0)	1 (1 to 1)	–0.55	.58
Postoperative AST	0 (0 to 0)	1 (1 to 1)	–0.55	.58
Postoperative BUN	0 (–0.01 to 0)	1 (0.99 to 1)	–1.09	.28
Postoperative Cr	0 (0 to 0)	1 (1 to 1)	1.18	.24
Group	–0.06 (–0.08 to –0.04)	0.94 (0.92 to 0.96)	–2.62	.01
TTR^h^	–0.14 (–0.19 to –0.09)	0.87 (0.83 to 0.91)	–2.85	.005
FTTR^i^	–0.2 (–0.25 to –0.14)	0.82 (0.78 to 0.87)	–3.66	<.001
Revisit to hospital due to secondary end point	0.91 (0.83 to 1)	2.49 (2.28 to 2.73)	10.31	<.001
Death due to secondary end point	0.9 (0.7 to 1.11)	2.47 (2 to 3.04)	4.33	<.001

^a^OR: odds ratio.

^b^BMI: body mass index.

^c^EF: ejection fraction.

^d^ALT: alanine transaminase.

^e^AST: aspartate transaminase.

^f^BUN: blood urea nitrogen.

^g^Cr: creatinine.

^h^TTR: time in the therapeutic range.

^i^FTTR: fraction of time in therapeutic range.

## Discussion

In this study, there was a significantly higher level of TTR in the population that was administered with the internet-based anticoagulation management than in the population treated with conventional warfarin management (0.53 vs 0.46; *P*<.001) in a real-world medical setting. Management of warfarin anticoagulation by a specialized, telemedicine–based service was able to substantially improve the quality of anticoagulation therapy. Our research findings showed that the new telemedical warfarin management had lower incidences of general bleeding events (22/260, 6.11% vs 40/361, 11.08%; *P=*.02), all bleeding events (24/360, 6.67% vs 44/361, 12.19%; *P=*.01), and all bleeding and embolic events than did the conventional group. In addition, single factor logistic regression of TTR and all bleeding and embolic events demonstrated the superiority of the new internet-based warfarin management as well as the relationship between TTR and anticoagulation complications in the 2 groups.

Patients who receive mechanical heart valve replacement need lifelong anticoagulation therapy, and their INR is conventionally measured to adjust the anticoagulation strength and the dose of anticoagulation drugs. However, adjusting the warfarin dosage is a challenging task since there is heterogeneity in patients’ warfarin dosing [23]. It has been reported that Chinese patients require a lower dose of warfarin than do White patients although the intensity of the anticoagulation is comparable [24,25]. As Chinese patients have a lower warfarin requirement, there are concerns about whether the target INR range for Western populations also provides optional anticoagulation in Chinese patients. In one study, You et al concluded that an INR of 1.8 to 2.4 appeared to be associated with the lowest incidence of major bleeding or thromboembolic events in a cohort of Chinese patients receiving warfarin therapy for moderate-intensity anticoagulation [26]. Unfortunately, there is no guideline for the target value of INR in China. At the seminar before the start of the project, after rigorous discussion, the researchers from all subcenters finally determined the target INR window of 1.8 to 2.5 (1.8-2.2 for aortic valve replacement, 2.0-2.5 for mitral valve replacement, and 2.0-2.5 for a double valve replacement) according to the actual clinical situation and previous studies in our centers [23,27-30]. The effectiveness of warfarin anticoagulation therapy is usually expressed as TTR. Due to the relatively narrow INR window, the TTR in the 2 groups was correspondingly lower than that reported in other warfarin management studies [19,20,31,32].

The finding of high levels of TTR and low incidences of anticoagulation complications in the internet-based management group might be, at least in part, attributable to the education, reminders, and convenient doctor-patient communication of the new management platform, which has been described previously [16,22]. Led by the director of surgery and including surgeons, trained physician assistants, staff nurses, and pharmacists, the new anticoagulation management model was developed and combined health science promotion and education, portable coagulation indicator monitoring, warfarin-related gene monitoring, warfarin dosage predication, access to a professional outpatient clinic, and a publicity manual. Unlike other studies in which only the patients use the app, our mobile apps are specific to doctors and patients. The app for doctors was distributed to cardiovascular surgeons from the collaborating units participating in the study by the lead unit of this study; the app for patients was distributed to patients enrolled in this study by doctors participating in this study. The doctor’s app contained a patient management module and a professional learning module that allowed timely patient contact. The patient’s app contained the patients’ personal information, including hospitalization information and discharge matters requiring attention. The patient’s app was mainly divided into a health management module, doctor consultation module, and medical science popularization module. The app regularly reminded the patients of the time of the INR test. If patients missed the test, the software would warn them. This new platform completed the collection of clinical data and provided reliable and quality follow-up services and warfarin management for patients undergoing lifelong warfarin coagulation.

Telemedicine, a term used interchangeably with telehealth, is the distribution of health-related services and information via electronic information and telecommunication technologies [33]. Over the past four decades, telemedicine has become an increasingly effective alternative to traditional medicine and has evolved into an integrated technology used in hospitals and clinics [34]. As the public becomes more adept at using the internet and smartphone in all aspects of daily life, evolving app in health care will change the way in which patients and doctors interact [35]. A few previous studies explored several internet-based warfarin management approaches and showed the advantages of the new anticoagulation model [32,36-38]. However, their conclusions were not completely consistent.

Based on the modern communication technologies, a new type of anticoagulation mode for cardiac surgery was established via the “Internet+Medical” management mode, supported by knowledge-based clinical medical diagnosis and treatment. This prospective, multicenter, randomized, open-label, controlled trial fills a deficit in research and adds to the solid evidence which exists regarding optimal strategies for patients with an extended period of warfarin anticoagulation. The new internet-based anticoagulation management model has the potential to provide considerable social and economic benefits in terms of further improving the long-term survival and quality of life of patients undergoing lifelong anticoagulation therapy.

There were also some limitations to this study. First, for the anticoagulation complications, the sample size was relevantly small, and the results may not be representative of all populations with anticoagulation therapy; thus, more prospective, large sample, randomized studies are needed to confirm our findings. Second, patients’ age, education level, and disease severity might have affected their compliance, which also might have influenced the quality of anticoagulation after surgery. Third, we did not compare the cost in the 2 management groups or the difference between urban and rural patients. In future studies and follow-up research, we will compare the associated medical costs during follow-up and rehabilitation under the 2 warfarin management models.

Internet-based warfarin management is superior to conventional management, as it can reduce the anticoagulation complications in patients who receive long-term warfarin anticoagulation after mechanical heart valve replacement.

## Appendix

Multimedia Appendix 1 CONSORT-eHEALTH checklist (V 1.6.1).

## Abbreviations

INR: international normalized ratio
TTR: time in therapeutic range
